# Clinicopathologic characteristics and prognosis of gastroenteropancreatic neuroendocrine neoplasms: a multicenter study in South China

**DOI:** 10.1186/s40880-017-0218-3

**Published:** 2017-06-21

**Authors:** Cheng Fang, Wei Wang, Yu Zhang, Xingyu Feng, Jian Sun, Yujie Zeng, Ye Chen, Yong Li, Minhu Chen, Zhiwei Zhou, Jie Chen

**Affiliations:** 1 Department of Gastric Surgery, Sun Yat-sen University Cancer Center, State Key Laboratory of Oncology in South China, Collaborative Innovation Center for Cancer Medicine, 651 Dongfeng Road East, Guangzhou, 510060 Guangdong P. R. China; 2 Department of Gastroenterology, The First Affiliated Hospital of Sun Yat-sen University, Guangzhou, 510080 Guangdong P. R. China; 3 Department of General Surgery, Guangdong General Hospital, Guangdong Academy of Medical Science, Guangzhou, 510080 Guangdong P. R. China; 4 Department of Biliopancreatic Surgery, The Sun Yat-sen Memorial Hospital of Sun Yat-sen University, Guangdong Provincial Key Laboratory of Malignant Tumor Epigenetics and Gene Regulation, Guangzhou, 510120 Guangdong P. R. China; 5 Department of Gastrointestinal Surgery, The Sun Yat-sen Memorial Hospital of Sun Yat-sen University, Guangzhou, 510120 Guangdong P. R. China; 6 Department of Gastroenterology, Nanfang Hospital of Southern Medical University, Guangdong Provincial Key Laboratory of Gastroenterology, Guangzhou, 510515 Guangdong P. R. China

**Keywords:** Gastroenteropancreatic neuroendocrine neoplasms, Surgery, Prognosis, China

## Abstract

**Background:**

Gastroenteropancreatic neuroendocrine neoplasms (GEP-NENs) are a heterogeneous group of rare tumors. Many issues in terms of epidemiologic features, pathogenesis, and treatment of GEP-NENs are still under discussion. Our study aimed to analyze the clinicopathologic characteristics and prognosis of Chinese patients with GEP-NENs.

**Methods:**

Complete clinicopathologic data and survival information of 1183 patients with GEP-NENs treated between 2005 and 2015 were collected from five medical centers in Guangdong Province, China. Patient survival was estimated using the Kaplan–Meier method and analyzed using the log-rank test; prognostic factors were analyzed using the Cox proportional hazards model.

**Results:**

The most common tumor location was the rectum (37.4%), followed by the pancreas (28.1%), stomach (20.7%), small intestine (7.2%), appendix (3.4%), and colon (3.3%). After initial definitive diagnosis, 1016 (85.9%) patients underwent surgery. The 1-, 3-, and 5-year overall survival (OS) rates for the entire cohort were 87.9%, 78.5%, and 72.8%, respectively. The 3-year OS rates of patients with G1, G2, and G3 tumors were 93.1%, 82.7%, and 43.1%, respectively (*P* < 0.001). The 3-year OS rates of patients with stage I, II, III, and IV tumors were 96.0%, 87.3%, 64.0%, and 46.8%, respectively (*P* < 0.001). Patients with distant metastasis who underwent palliative surgery had a longer survival than those who did not (*P* = 0.003). Similar survival benefits of palliative surgery were observed in patients with neuroendocrine tumor (*P* = 0.031) or neuroendocrine carcinoma (*P* = 0.046). In multivariate analysis, age, grade, N category, M category, and surgery were found to be independent prognostic factors.

**Conclusions:**

Patients with GEP-NENs who are women, younger than 50 years old, have smaller tumor size, have lower tumor grade, have lower T/N/M category, and who undergo surgery can have potentially longer survival time. Our data showed that surgery can improve the prognosis of GEP-NEN patients with distant metastasis. However, randomized controlled trials need to be conducted to establish the optimal criteria for selecting patients to undergo surgery.

## Background

Neuroendocrine neoplasms (NENs) are a heterogeneous group of rare tumors with different and complex clinical behaviors, originating from peptidergic neurons and neuroendocrine cells throughout the body, and most are gastroenteropancreatic neuroendocrine neoplasms (GEP-NENs) [1]. Over the years, the nomenclature and classification of NENs have undergone significant changes. In 1907, Oberndorfer [2] first described it as a benign “carcinoid” tumor. In 2000, the term “neuroendocrine tumors” was officially used in the World Health Organization (WHO) classification to more accurately depict their malignant potential. In 2010, the Ki-67 proliferative index and mitotic count were first used in the WHO classification as important diagnostic and prognostic factors for NENs [3]. The current staging systems developed by the American Joint Committee on Cancer/Union for International Cancer Control (AJCC/UICC) and European Neuroendocrine Tumor Society (ENETS) are both based on the TNM staging system.

To data, only a few studies, based on data from national cancer registries—mainly from the United States [4], Norway [5], England [6], Spain [7], Germany [8], and Korea [9]—have showed epidemiologic features of NENs. However, for some of these studies, the current staging system and diagnostic criteria were not used, or detailed demographic and clinicopathologic characteristics or therapeutic intervention information were lacking. Generally, the incidence of GEP-NENs has increased continuously worldwide over the last decades. In patients from Western countries, primary tumors locate mostly in the small intestine, rectum, and pancreas [4–7]. Furthermore, the distribution of tumor stages and patients’ overall survival (OS) rates are discrepant in different countries. Although several single-center studies with limited sample size on the clinicopathologic features of GEP-NENs have been performed in Chinese populations [10, 11], a multicenter study with a large population has been needed to fully understand this rare tumor and identify optimal therapeutic strategies. In 2010, several large centers in Guangdong Province, China, began a long-term collaboration focused on providing multidisciplinary treatments of NENs. The present study aimed to analyze clinicopathologic characteristics and prognosis of GEP-NEN patients from these centers in South China.

## Patients and methods

### Patient selection

We collected data from the case management systems of five medical centers in Guangdong Province, China: Sun Yat-sen University Cancer Center, the First Affiliated Hospital of Sun Yat-sen University, Guangdong General Hospital, Nanfang Hospital of Southern Medical University, and Sun Yat-sen Memorial Hospital of Sun Yat-sen University. We included only patients who (1) were histopathologically diagnosed with GEP-NENs; (2) were treated and followed between January 2005 and December 2015; and (3) had complete medical records containing demographic data, clinicopathologic data, and follow-up results. Patients with additional synchronous or metachronous malignancies were excluded.

The present study was approved by the ethics committees of the five hospitals and complied with the Declaration of Helsinki.

### Diagnosis, staging, and treatment

GEP-NENs were histopathologically defined according to the current WHO 2010 classifications [3]; for patients diagnosed and treated before 2010, tumors were re-defined. Functional or nonfunctional GEP-NENs were defined according to whether the patients presented with clinical symptoms caused by hormones. The tumor locations of GEP-NENs were categorized as the stomach, pancreas, small intestine (including the duodenum, jejunum, and ileum), appendix, colon, and rectum. Pathologic examinations of the sections from endoscopic biopsies, intraoperative incisional biopsies, and resected gross specimens were performed by pathologists following diagnostic criteria which contained typical morphological findings and immunohistochemical staining of neuroendocrine markers, including chromogranin A and synaptophysin. Based on the WHO 2010 classifications [3], G1, G2, and G3 grading levels were classified according to the Ki-67 index (≤2%, 3%–20%, and >20%) and mitotic rates (<2 per 10 HPF [high power field], 2–20 per 10 HPF, and >20 per 10 HPF). If the grade of Ki-67 index was not in agreement with the grade of mitotic rate, the parameter with the higher grade was used for classification. According to different degrees of pathologic differentiation, all NENs were divided into well-differentiated neuroendocrine tumors (NETs), poorly differentiated neuroendocrine carcinomas (NECs), and mixed adenoneuroendocrine carcinomas (MANECs) which contained two components (adenocarcinoma and neuroendocrine neoplasms). Based on clinical, pathologic, and imaging data, the tumors were classified into stages I, II, III, and IV according to the ENETS TNM classification [12, 13]. Therapeutic modalities included surgeries such as endoscopic resection (endoscopic mucosal resection or endoscopic submucosal dissection), radical resection, and debulking or cytoreductive surgery and systematic therapies such as chemotherapy and somatostatin analogues or targeted drugs.

### Follow-up

Follow-up data were based on outpatient records and on messaging and/or telephonic interviews conducted between the day patients were discharged and March 31, 2016. A strict program of disease monitoring including electronic gastroduodenoscopic examinations, abdominal ultrasonography (and computed tomography, if necessary), chest X-rays, and blood examinations, was conducted, if clinically required. Recommended follow-up intervals were 6 months. OS was defined as the duration from the date of pathologic diagnosis to the date of death or the last follow-up.

### Statistical analysis

Data were analyzed using the SPSS software version 18.0 (SPSS Inc., Chicago, IL, USA). Categorical variables were analyzed using the Chi-square test. Kaplan–Meier analysis was used to estimate the cumulative OS rate; log-rank test was used to analyze significances among the different groups; and the Cox proportional hazards model was used for multivariate analysis. The two-tailed *P* values less than 0.05 were considered statistically significant.

## Results

### Demographic data

A total of 1496 patients were included. Of these, 1183 patients were selected into our study, since 105 patients were lost during follow-up, 182 did not have complete clinicopathologic data, and 26 had other types of malignancies and were thus excluded. The selected patients included 482 (40.7%) from Sun Yat-sen University Cancer Center, 309 (26.1%) from the First Affiliated Hospital of Sun Yat-sen University, 143 (12.1%) from Guangdong General Hospital, 131 (11.1%) from Nanfang Hospital of Southern Medical University, and 118 (10.0%) from Sun Yat-sen Memorial Hospital of Sun Yat-sen University. There were 715 men and 468 women in a ratio of 1.5:1, with a median age of 51 years (range, 9–87 years).

### Clinicopathologic factors

Clinicopathologic characteristics of these 1183 GEP-NEN patients are shown in Table 1. Nonfunctional tumors accounted for 82.8% (979) of GEP-NENs; the remaining 17.2% (204) were functional tumors. The most common tumor location was the rectum, followed by the pancreas, stomach, small intestine, appendix, and colon. The median diameter of the primary tumor was 2.0 cm (range, 0.1–20.0 cm), based on postoperative resection specimens or imaging examinations before surgery. Among all cases, 608 (51.4%) were G1, 270 (22.8%) were G2, and 305 (25.8%) were G3. Well-differentiated NETs accounted for 74.5% (881). Based on ENETS criteria, 479 cases (40.5%) were stage I, 246 (20.8%) were stage II, 200 (16.9%) were stage III, and 258 (21.8%) were stage IV. In this study, 85.9% (1016) of the patients underwent surgery (radical or palliative resection). In addition, systematic treatment was administered to 306 (25.9%) patients. Chi-square analyses for the variables in the surgery and non-surgery groups of patients with M1 category tumors showed that the clinicopathologic characteristics between the two groups were not significantly different, except for tumor size and location (Table 2). In the surgery group, more patients had tumors located in the stomach and intestine and with tumor sizes smaller than or equal to 4.0 cm compared with the non-surgery group.

**Table 1:** Clinicopathologic characteristics of 1183 patients with gastroenteropancreatic neuroendocrine neoplasms (GEP-NENs)

Variable	No. of cases (%)
Gender	
Female	468 (39.6)
Male	715 (60.4)
Age (years)	
≤50	564 (47.7)
>50	619 (52.3)
Tumor functionality	
Nonfunctional	979 (82.8)
Functional	204 (17.2)
Tumor location	
Stomach	245 (20.7)
Pancreas	332 (28.1)
Small intestine	85 (7.2)
Appendix	40 (3.4)
Colon	39 (3.3)
Rectum	442 (37.4)
Tumor size (cm)	
<2.0	616 (52.1)
2.0–4.0	272 (23.0)
>4.0	295 (24.9)
Grade (WHO 2010 classification)	
G1	608 (51.4)
G2	270 (22.8)
G3	305 (25.8)
Tumor type	
NET	881 (74.5)
NEC	261 (22.1)
MANEC	41 (3.5)
T category (ENETS criteria)	
T1	510 (43.1)
T2	220 (18.6)
T3	314 (26.5)
T4	139 (11.7)
N category (ENETS criteria)	
N0	846 (71.5)
N1	337 (28.5)
M category (ENETS criteria)	
M0	925 (78.2)
M1	258 (21.8)
TNM stage (ENETS criteria)	
I	479 (40.5)
II	246 (20.8)
III	200 (16.9)
IV	258 (21.8)
Surgery	
Yes	1016 (85.9)
No	167 (14.1)

*WHO* World Health Organization, *NET* neuroendocrine tumor, *NEC* neuroendocrine carcinoma, *MANEC* mixed adenoneuroendocrine carcinoma, *ENETS* European Neuroendocrine Tumor Society

**Table 2:** Chi-square analysis for clinicopathologic variables in patients with M1 category GEP-NENs who did or did not undergo surgery

Variable	Surgery group [cases (%)]	Non-surgery group [cases (%)]	*P* value
Total	127	131	
Gender			0.233
Male	93 (73.2)	87 (66.4)	
Female	34 (26.8)	44 (33.6)	
Age (years)			0.403
≤50	52 (40.9)	47 (35.9)	
>50	75 (59.1)	84 (64.1)	
Tumor functionality			0.144
Nonfunctional	112 (88.2)	107 (81.7)	
Functional	15 (11.8)	24 (18.3)	
Tumor location			<0.001
Stomach	39 (30.7)	29 (22.1)	
Pancreas	33 (26.0)	70 (53.4)	
Small intestine	17 (13.4)	8 (6.1)	
Appendix	1 (0.8)	0 (0.0)	
Colon	10 (7.9)	4 (3.1)	
Rectum	27 (21.2)	20 (15.3)	
Tumor size (cm)			0.006
<2.0	25 (19.7)	14 (10.7)	
2.0–4.0	50 (39.4)	38 (29.0)	
>4.0	52 (40.9)	79 (60.3)	
Grade			0.743
G1	25 (19.7)	24 (18.3)	
G2	46 (36.2)	43 (32.8)	
G3	56 (44.1)	64 (48.9)	
Tumor type			0.574
NET	70 (55.1)	65 (49.6)	
NEC	52 (40.9)	62 (47.3)	
MANEC	5 (3.9)	4 (3.1)	

*NET* neuroendocrine tumor, *NEC* neuroendocrine carcinoma, *MANEC* mixed adenoneuroendocrine carcinoma

### Patient survival

**Fig. 1:**
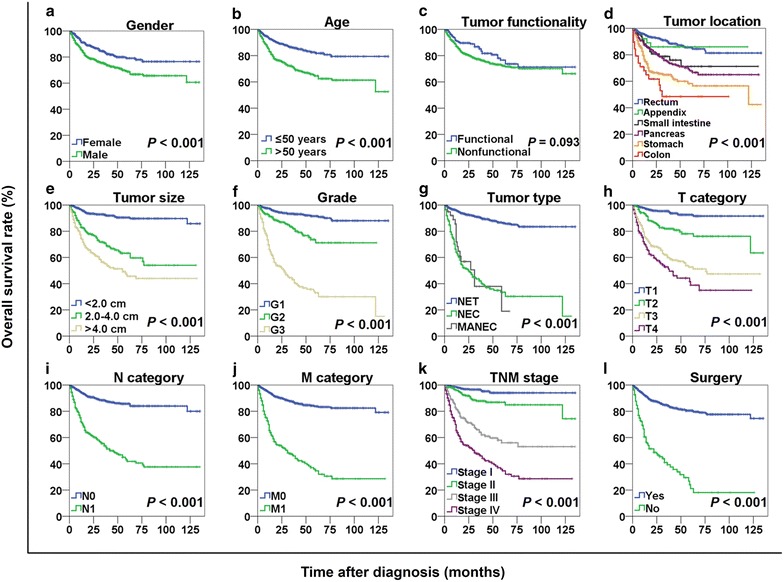
Overall survival (OS) curves stratified by different variables in 1183 patients with gastroenteropancreatic neuroendocrine neoplasms (GEP-NENs). OS curves stratified by gender (**a**), age (**b**), tumor functionality (**c**), tumor location (**d**), tumor size (**e**), tumor grade (**f**), tumor type (**g**), T category (**h**), N category (**i**), M category (**j**), TNM stage (**k**), and surgery (**l**). OS was different among subgroups stratified by all variables (all *P* < 0.001) except tumor functionality (*P* = 0.093). *NET* neuroendocrine tumor, *NEC* neuroendocrine carcinoma, *MANEC* mixed adenoneuroendocrine carcinoma

**Fig. 2:**
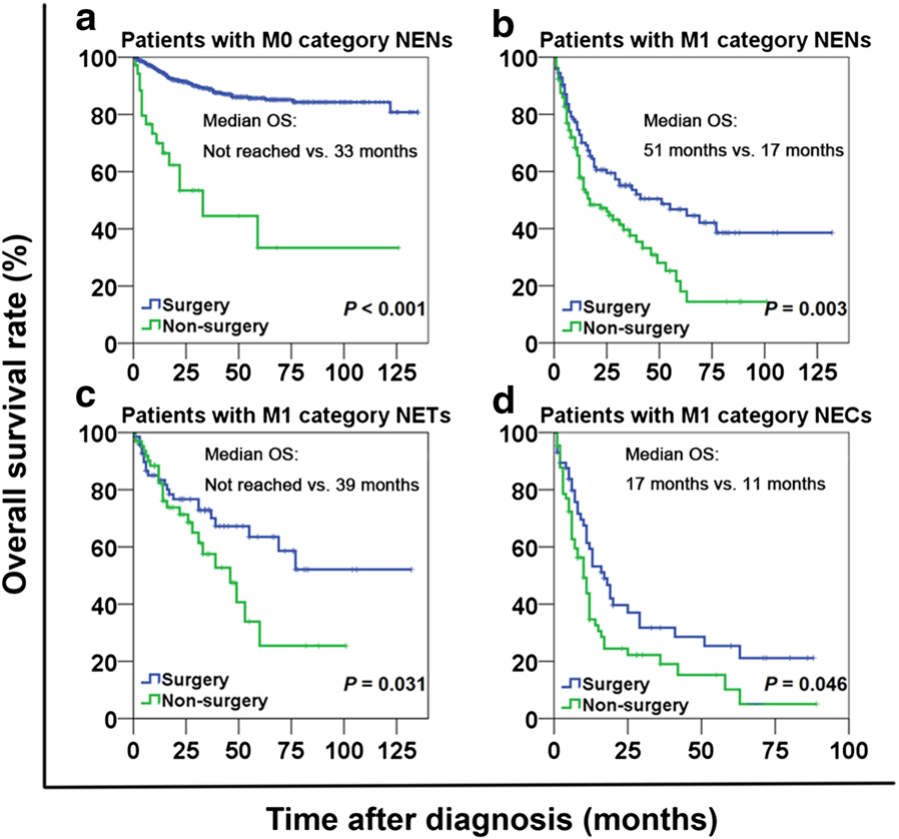
Patients with neuroendocrine neoplasms (NENs) who underwent surgery had longer OS than those who did not. **a** OS curves stratified by treatment in patients with M0 category NENs. **b** OS curves of patients with M1 category NENs. **c** OS curves of patients with M1 category NETs. **d** OS curves of patients with M1 category NECs. In the above subgroups, OS is always significantly longer in patients who underwent surgery than in those who did not (all *P* < 0.05)

The median OS for GEP-NEN patients was 28 months (range, 4–135 months). The 1-, 3-, and 5-year OS rates were 87.9%, 78.5%, and 72.8%, respectively. The OS curves stratified by gender, age, tumor functionality, tumor location, tumor size, grade, tumor type, T category, N category, M category, TNM stage, and treatment are displayed in Fig. 1. The 3-year OS rates for patients with TNM stage I, II, III, and IV diseases were 96.0%, 87.3%, 64.0%, and 46.8%, respectively (*P* < 0.001). The 3-year OS rates for patients with G1, G2, and G3 diseases were 93.1%, 82.7%, and 43.1%, respectively (*P* < 0.001). With respect to tumor locations, the 3-year OS rates for patients with tumors in the rectum, appendix, small intestine, pancreas, stomach, and colon were 90.2%, 86.0%, 75.8%, 75.3%, 64.6%, and 48.5%, respectively (Table 3). The 3-year OS rate of patients with M0 category tumors who underwent surgery was higher than that of those who did not (88.7% vs. 45.2%, *P* < 0.001) (Fig. 2a). Interestingly, patients with M1 category tumors who underwent palliative surgery had a longer median OS duration compared with those who did not undergo palliative surgery (51 months vs. 17 months, *P* = 0.003) (Fig. 2b). Furthermore, stratified analyses showed similar survival benefits for both patients with NETs (median OS: not reached vs. 39 months, *P* = 0.031) (Fig. 2c) and patients with NECs (median OS: 17 months vs. 11 months, *P* = 0.046) (Fig. 2d).

**Table 3:** Univariate and multivariate analyses of prognostic factors for overall survival of 1183 patients with GEP-NENs

Variable	3-year OS rate (%)	Univariate analysis		Multivariate analysis				
		Log-rank χ^2^	*P*	B	SE	HR	95% CI	*P*
Gender		15.660	<0.001*	0.194	0.150	1.190	0.905–1.629	0.196
Female	84.1							
Male	74.9							
Age (years)		38.828	<0.001*	0.315	0.148	1.367	1.025–1.833	0.034
≤50	86.1							
>50	71.4							
Tumor functionality		2.821	0.093					ND
Nonfunctional	77.1							
Functional	85.2							
Tumor location		82.903	<0.001*					0.460
Stomach	64.6			NA	NA	1	Reference	NA
Pancreas	75.3			0.297	0.288	1.070	0.766–2.366	0.301
Small intestine	75.8			0.264	0.203	1.699	0.875–1.939	0.192
Appendix	86.0			0.456	0.279	1.665	0.913–2.726	0.103
Colon	48.5			0.216	0.213	1.227	0.817–1.883	0.311
Rectum	90.2			0.697	0.494	2.988	0.762–5.292	0.159
Tumor size (cm)		170.024	<0.001*					0.174
<2.0	92.6			NA	NA	1	Reference	NA
2.0–4.0	71.0			0.082	0.247	1.061	0.669–1.760	0.740
>4.0	55.7			0.364	0.263	1.411	0.859–2.408	0.167
Grade (WHO 2010 classification)		325.954	<0.001*					<0.001
G1	93.1			NA	NA	1	Reference	NA
G2	82.7			0.379	0.229	1.489	0.933–2.287	0.097
G3	43.1			1.627	0.221	5.172	3.301–7.837	<0.001
Tumor type		336.619	<0.001*					ND
NET	90.6							
NEC	42.1							
MANEC	37.9							
T category (ENETS criteria)		204.865	<0.001*					0.401
T1	94.7			NA	NA	1	Reference	NA
T2	81.6			0.190	0.307	1.189	0.663–2.206	0.536
T3	60.1			0.454	0.308	1.594	0.862–2.878	0.140
T4	51.3			0.456	0.344	1.563	0.804–3.097	0.185
N category (ENETS criteria)		189.400	<0.001*	0.430	0.156	1.552	1.132–2.086	0.006
N0	88.2							
N1	53.8							
M category (ENETS criteria)		255.266	<0.001*	0.784	0.164	2.107	1.590–3.018	<0.001
M0	87.2							
M1	46.8							
TNM stage (ENETS criteria)		332.157	<0.001*					ND
I	96.0							
II	87.3							
III	64.0							
IV	46.8							
Surgery		212.543	<0.001*	0.521	0.175	1.730	1.195–2.370	0.003
Yes	84.4							
No	39.3							

*OS* overall survival, *B* beta value, *SE* standard error, *HR* hazard ratio, *CI* confidence interval, *ND* not detected, *NA* not applicable

* Variables with *P* value less than 0.05 entered into multivariate analyses, except for tumor type and TNM stage

### Univariate and multivariate analyses

To determine the prognostic factors of OS, multivariate analysis using the Cox proportional hazards model with a forward stepwise model was performed for the 1183 cases of GEP-NENs. Gender, age, tumor functionality, tumor location, tumor size, grade, tumor type, T category, N category, M category, TNM stage, and treatment were included in the univariate analysis (Table 3). The results showed that all the above factors, except for tumor functionality, were significantly associated with prognosis. Moreover, when these significant variables were entered into the Cox proportional hazards model, age, grade, N category, M category, and treatment were found to be independent prognostic factors (Table 3).

## Discussion

In this study, we collected and analyzed the clinicopathologic features of 1183 GEP-NEN patients with complete follow-up data from five large hospitals in South China. Our data showed the most common locations of primary tumor were the rectum, pancreas, and stomach. By multivariate analysis, age, grade, N category, M category, and treatment were found to be independent prognostic factors. Of note, our findings have shown that patients with distant metastasis who underwent palliative surgery had a longer survival time than those who did not undergo palliative surgery.

Until recently, only four studies containing more than 1000 GEP-NEN cases were reported worldwide [5, 6, 14, 15]. As with other studies [15–17], we reported the gender ratio (male vs. female, 1.5:1) and median age (51 years) at diagnosis; and found most of the GEP-NENs in our study were nonfunctional and were found by incidental diagnosis, physical examination, or nonspecific symptoms, such as abdominal pain, gastrointestinal bleeding, diarrhea, and jaundice, which are similar to previous studies [18, 19]. The distribution of tumor locations of GEP-NENs that showed in our study was similar to those found in several other Asian studies [9, 11]. In most reports from the United States and European countries—typically those with data derived from the National Cancer Institute’s Surveillance, Epidemiology and End Results (SEER) database [4], the National Cancer Registry of Gastroenteropancreatic Neuroendocrine Tumors (RGENE) [6], and the Norwegian Registry of Cancer (NRC) [5] —the rectum, small intestine, and pancreas were the most common NEN locations, whereas gastric NENs accounted for less than 10% of GEP-NEN cases. However, in the present study, the rectum, pancreas, and stomach were the most common locations of NENs, whereas small intestine NENs accounted for less than 10%. We consider that racial disparities may contribute to the distinctions in distributions of tumor locations. Furthermore, the inconsistencies may also be due to the high incidence of gastric cancer in China [20, 21], which has raised health awareness and encouraged more cautious people to have regular physical examinations to identify gastric diseases. All specimens in the current analysis were reviewed and graded according to the latest nomenclature and classification system for GEP-NENs. In our study, G1 tumors accounted for 51.4%, which was consistent with the results of studies from Korea [9], Austria [22], and the Netherlands [23]. Based on the ENETS staging system, lymph node and/or distant metastasis occurred in 38.7% of patients, which was lower than the occurrence rates reported in Spain (44.2%) by Garcia-Carbonero et al. [7] and Hong Kong, China (53.4%) by Rothenstein et al. [24]. The 5-year OS rate in our entire cohort was 72.8%, which was higher than those in cohorts based on the SEER registries from the United States (50%) [4] and NRC registries from Norway (59%) [5], and similar to those in some cohorts from European countries (75%–79%) [6–8]. The discrepancy with data from SEER and NRC may be attributed to the high ratio of NEC patients in the SEER database [4] as well as racial and geographic differences in the NRC database [5]. More studies across different continents and countries to compare these clinicopathologic characteristics and prognosis may be required to better understand GEP-NENs.

In our univariate analysis, women and younger patients with smaller tumors, lower tumor grade, less invasion, and surgical treatment and those without lymph node or distant metastasis had significantly longer OS (all *P* < 0.001). Gender was not a prognostic factor in several studies [5–8], and only one study showed a survival benefit for female patients [17]. Interestingly, in our study, patients with distant metastasis who underwent palliative surgery had a longer OS than those who did not undergo palliative surgery (median OS: 51 months vs. 17 months, *P* = 0.003). Our results showed that many patients with distant metastasis whose tumors were located in the stomach and intestine and who had tumor sizes less than or equal to 4 cm underwent surgery. This phenomenon may be partly due to higher rates of preoperative symptoms, such as massive gastrointestinal bleeding and obstruction, which required emergent surgery. Also, compared with pancreatic surgeries, gastrointestinal surgeries showed lower rates of postoperative complications [25–27]. Moreover, patients with smaller tumor sizes were possibly considered to have lower tumor burden and were more likely to undergo surgery. Different treatments were selected in consideration of discrepancies of tumor location and size, which may account for the survival difference among patients with distant metastasis. In addition, analysis stratified by tumor types showed that patients with distant metastasis of NETs (similar to G1/G2 tumors with distant metastasis) and NECs (similar to G3 tumors with distant metastasis) could benefit from surgery (*P* = 0.031 and *P* = 0.046, respectively), which has not been reported in any other study. On the one hand, ENETS guidelines recommend that debulking surgery may be considered for patients with nonfunctional and advanced-stage NETs if the disease does not progress over 6 months and for patients who are suffering from symptoms related to tumor burden [18]. On the other hand, in the context of patients with advanced metastatic NECs, debulking or cytoreductive surgery of primary tumors and surgical resection of metastases are not recommended [18]. Other ablative strategies for liver metastasis are also discouraged [18]. This means that surgery may be important for strictly selected patients with advanced-stage NECs, such as patients with low tumor burden and limited distant metastases. The survival benefits showed in our study might also be explained by the fact that most advanced-stage cases were comprehensively reviewed by a multidisciplinary team which may select patients with lower metastatic tumor burden and relatively longer stable disease period or patients with better physical condition. Nevertheless, since this study included a limited number of patients with stage IV disease, prospective randomized controlled trials are needed to validate the benefit from surgery and to determine the time point for surgery in patients with advanced-stage disease in clinical practice.

Our study had several limitations. First, data from the five large hospitals cannot be representative of the entire country of China. However, each of these hospitals has different sets of patients. For example, patients with a tumor mass would probably go to the specialized cancer center; those with mild symptoms or who have normal physical examinations would probably go to one of the general hospitals. Thus, because of the inclusion of patients with different characteristics, results of this multicenter study may be representative. Second, since GEP-NENs are rare heterogeneous tumors, case numbers in specific subgroups such as patients who undergo different surgical and systematic treatments, are limited, and data could not be statistically analyzed. Third, since the diseases of most patients were diagnosed in the past 5 years, the follow-up duration in our study may be not long enough because these patients usually have a better prognosis compared with patients with other malignancies.

## Conclusions

Our results showed that surgery increased the survival of patients with distant metastasis, even of patients with NECs. However, establishing the optimal criteria to select advanced patients to undergo surgery in clinical practices requires high-level evidence from randomized controlled trials.
